# Feedback Enhances Preschoolers’ Performance in an Inhibitory Control Task

**DOI:** 10.3389/fpsyg.2019.00977

**Published:** 2019-05-08

**Authors:** Niamh Oeri, David Buttelmann, Annik E. Voelke, Claudia M. Roebers

**Affiliations:** Department of Developmental Psychology, Institute of Psychology, University of Bern, Bern, Switzerland

**Keywords:** goal maintenance, feedback, inhibition, executive function, child development

## Abstract

Accomplishing inhibition tasks requires not only inhibitory skills but also goal maintenance. The present study aimed to disentangle goal maintenance from inhibition. Therefore, we experimentally manipulated goal-maintenance demands by means of feedback. Three-year-old (*n* = 84) and 4-year-old (*n* = 75) preschoolers were randomly assigned to one of four experimental conditions. Results revealed an age-dependent pattern: three-year-olds that were assigned to one of the conditions with feedback outperformed those assigned to the control condition without feedback. It seems that especially performance-related feedback reduced goal-maintenance demands in 3-year-olds, resulting in enhanced inhibitory performance. Four-year-olds, in contrast, showed high performance across all conditions. Age-differences between the 3- and 4-year-olds were only significant for the control condition. Thus, with feedback, performance of the 3-year-olds was similar to that of the 4-year-olds. The present results seem to indicate that in an inhibition task, 3-year-olds’ struggle not only with inhibiting a prepotent response but also with adhering to the task goal.

## Introduction

Demands on inhibition are ubiquitous in a child’s life. Following instructions in school as well as sharing toys with a sibling or a peer demand inhibition. Inhibition refers to the ability not only to resist interference from distracting stimuli, but also to suppress prepotent responses. Along with shifting and working memory, inhibition is one of the primary components of executive functions (EF; Miyake et al., 2000). Research has shown that inhibition develops rapidly during preschool years (Zelazo et al., 2003; Carlson, 2005). Beyond that, experimental work has focused on manipulating inhibitory demands across different cognitive tasks. Whenever inhibition demands were reduced, task performance increased. Similar findings have been found across different inhibition tasks, such as the Day-night task (Diamond et al., 2002), different versions of the Simon task (Davidson et al., 2006) or the Car task (Buttelmann and Berger, 2019). However, Marcovitch et al. (2007) argue that developmental theories tend to neglect the importance of goal maintenance for EF skills such as inhibition. Goal maintenance refers to the active representation of the task rule or goal state and plays a critical role in EF tasks. It involves the ability to represent the goal and hold it active to guide behavior accordingly (Marcovitch et al., 2007, 2010). Hence, a preschoolers’ improvement in an inhibition task might be due to developmental progress in goal maintenance and not necessarily solely related to inhibition development. By means of an experimental research design, we aimed to disentangle goal maintenance from inhibition to gain a better understanding of preschoolers’ inhibitory skills.

Studies with school-aged children provide supporting evidence for goal maintenance being involved in the process of solving inhibition tasks. For example, in an experimental study, younger participants showed weaker performance in a stroop task compared to older ones (Bub et al., 2006). Subsequent analyses of the response time distributions showed that weaker performance was due to difficulties in maintaining the task rule and could not be explained by inefficient inhibition. Additionally, a correlational study showed that preschoolers’ inhibitory performance was related to failure in goal maintenance (Towse et al., 2007).

Both, theoretical considerations (Marcovitch et al., 2007) and empirical findings (Bub et al., 2006) suggest that goal maintenance plays a critical role when solving an inhibition task. Furthermore, there is experimental work manipulating goal-maintenance demands with visual cues (e.g., Chevalier and Blaye, 2008; Blaye and Chevalier, 2011). Goal-maintenance demands were varied by means of cue transparency (arbitrary cues vs. transparent). While arbitrary cues are largely unrelated to the task goal, transparent cues are highly associated with the task goal. Consequently, transparent cues reduce demands on goal-maintenance for the individual. Results showed that 4-year-olds’ inhibition and switching performance was better with transparent cues compared to arbitrary cues. Similar findings were found for different EF tasks, such as the Shape school task as well as the DCCS task (Chevalier and Blaye, 2008; Blaye and Chevalier, 2011). Beyond that, work with the Flanker task revealed congruent findings. Although the study by Rueda et al. (2004) was originally set up to examine a different research question, results revealed that inhibitory performance was better in trials with a valid cue appearing before the target stimulus, compared to trials without valid cues. Thus, there is evidence that once goal maintenance demands are reduced, inhibitory performance increases; most likely because attentional processes are underlying this relation (Rueda et al., 2004; Marcovitch et al., 2007). In everyday situations, however, it is less common to receive direct cues facilitating a certain behavior. Children are more likely to receive an indirect signal in response to a certain behavior, most likely some kind of feedback. However, so far, experimental approaches only involved direct manipulations of goal-maintenance. Therefore, the current study aimed to extend previous experimental approaches by manipulating goal maintenance *indirectly*, by means of feedback.

Previous work with preschoolers revealed beneficial effects of feedback for switching performance in the dimensional chance cart sorting task (DCCS). While Bohlmann and Fenson (2005) were able to show that preschoolers’ switching performance increased when receiving corrective feedback, van Bers et al. (2014) work underlines preschoolers’ sensitivity to feedback quality. The study showed that a causal relation between response behavior and feedback was crucial for lasting feedback effects on switching performance. One could argue that feedback reminds participants of the task rule, thereby supporting an active representation of the task rule and goal state. Consequently, it could be that not only cues appearing before the target but also feedback related to performance reduces goal maintenance demands. Hence, if goal-maintenance demands could be successfully reduced trough feedback, performance would increase for conditions with feedback compared to conditions without feedback. An alternative explanation could be that feedback predominantly affects motivation (Dowsett and Livesey, 2000). That is, enhanced performance after receiving feedback would be due to greater motivation and not due to a reduction of goal-maintenance demands *per se*. An experimental research design that includes performance-related feedback as well as performance-unrelated feedback allows differing between the effects of reduced goal-maintenance demands and the effects of motivation on performance. An additional issue that needs careful consideration is the frequency of feedback itself. More specifically, constant feedback might evoke satiation effects, which in turn might affect performance negatively (Hattie and Timperley, 2007).

So far, most work on the effects of feedback has been conducted with the DCCS. Although inhibition is required in this sorting task, flexibility in switching between task rules is the particular skill assessed with the DCCS. Thus, it remains unexplored how feedback affects goal maintenance in non-sorting inhibition tasks, measuring inhibition more precisely. There are different inhibition tasks that can be administered in preschool age. However, the flanker task has three particular advantages: Firstly, the task is computerized and therefore quantifies effects precisely in terms of two different variables [reaction time (RT) and accuracy]. Secondly, the task does not require a verbal response. This eliminates confounding language-based influences (Mullane et al., 2009; Best and Miller, 2010). Thirdly, the task can be administered across different age groups, ranging from preschool age into adulthood (Rueda et al., 2004; Zelazo et al., 2013). Thus, extending previous research approaches, we aimed to examine if by means of feedback, goal-maintenance demands can be reduced when considering motivational effects.

### The Present Study

The aim of the present study was two-fold. Firstly, we aimed to examine if goal-maintenance demands can also be reduced for the flanker task. Therefore, we manipulated goal maintenance indirectly by means of feedback. In order to distinguish between (increased) motivation affecting task performance and (reduced) goal maintenance demands affecting task performance, we created four different experimental conditions: A control condition with no feedback throughout the task. Then there were two *performance-related feedback conditions*, for which the frequency of the occurring feedback differed (i.e., constant feedback, intermittent feedback). These two conditions were used to rule out possible satiation effects (Hattie and Timperley, 2007). The fourth condition was a *performance-unrelated feedback condition*. By contrasting effects of *performance-related feedback* to effects of *performance-unrelated feedback*, we were able to control for possible motivational effects on task performance. The second aim of the present study was to examine age effects. Since the preschool period is a sensitive window for inhibition development (Zelazo et al., 2003; Carlson, 2005), we were interested in possible differences in susceptibility to feedback between 3- and 4-year-olds.

## Materials and Methods

### Participants

A sample of 159 3-year olds (*n* = 84, mean age 42 months and 24 days, age range 3.0–3.11; 48% female) and 4-year-olds (*n* = 75, mean age 52 months and 21 days, age range 4.0–4.11; 56% female) participated in the study. The children were recruited and tested in day-care centers. Participants were predominately Caucasian from middle-class families, reflecting the characteristics of the local community. Written consent from the children’s parents as well as verbal assent from the child was obtained before testing. The ethics committee of the local faculty approved the study. Twelve additional participants were tested but had to be excluded, either due to technical problems (*n* = 4), loss of interest or motivation (*n* = 8).

### Materials

#### Flanker Task

Similar age-adapted versions of the Flanker task (Eriksen and Eriksen, 1974) have been used with preschoolers before (see e.g., Fatzer and Roebers, 2013; Zelazo et al., 2013). However, these studies had relatively high dropout rates (up to 25% of 3- to 6-year olds aborting the task). Piloting work with 3-year olds revealed that a familiarization phase before task instruction was crucial for task completion. During the familiarization phase, the child practiced pressing the response buttons (i.e., became acquainted with the motoric demands of the task). Establishing a relation between pressing the response button and external feedback reduced the dropout rate to 5%, irrespectively of the experimental condition. The computerized task (E-Prime Software, Psychology Software Tools, Pittsburgh, PA, United States) was presented on a laptop (12.1′′ screens). Two response buttons were placed in front of the child. As mentioned, before introducing the flanker task, the child was first familiarized with the response mode of the task (i.e., pressing the response buttons). During the familiarization phase, pressing the buttons was always followed by an acoustic feedback as well as a visual feedback on screen (i.e., clapping sound accompanied by a big yellow smiley). Then, the experimenter instructed participants to respond to the orientation of the centrally presented target (fish) by pressing the left or the right response button accordingly. In congruent trials, the target and the distractors (four flanking fish, two on each side of the central fish) were facing in the same direction. In incongruent trials, however, the target and the distractors were facing in opposite directions. After the instruction, participants completed two practice blocks (with four trials each). In the first practice block, correct answers were followed by positive acoustic feedback (i.e., a “clapping-sound”). Incorrect answers were followed by negative acoustic feedback (i.e., a “horn-sound”). If participants did not meet the practice criterion (i.e., at least three correct answers out of four), the first practice block was repeated (applied to 5.2% of the sample). As for the second practice block, no acoustic feedback was provided. Again, if participants did not meet the practice criterion (i.e., at least three correct answers out of four), the second practice block was repeated (applied to 4.7% of the sample).

The experimental block consisted of 24 trials (i.e., 12 congruent and 12 incongruent). One single experimental trial consisted of the following components: An inter-stimulus interval, randomly varying between 2000 and 3000 ms, was followed by a fixation cross. After 100 ms a yellow circle surrounded the fixation cross (400 ms) and an auditory signal (100 ms) occurred. In total, the fixation cross was presented for 500 ms. Then, the stimulus was presented until the child responded. There was no time limit to children’s responses. At the end of the experimental block (i.e., after all 24 trials), positive feedback (i.e., image of a big laughing fish accompanied by the “clapping sound”) was provided.

The single trial components were identical in all four conditions. However, feedback quality differed across the four conditions (for an overview see Table 1). There was one performance-unrelated feedback condition and one control condition without any feedback. The two performance-related feedback conditions were the intermittent feedback condition and the constant feedback condition. As for the intermittent feedback condition, a positive, acoustic feedback (i.e., clapping sound) was provided after the 3rd, 5th, 10th, 13th, 18th, and 21st correct answer. As for the constant feedback condition, a positive, acoustic feedback (i.e., clapping sound) was provided after every correct answer. Further, for the performance-unrelated constant feedback condition, a background melody accompanied the task and every response, regardless of its correctness, was followed by an entertaining sound (i.e., “noise” of a fish swimming away). Finally, for the fourth condition, the control condition, no feedback was provided between the trials.

**Table 1:** Overview Experimental Conditions.

Condition	Feedback type	Description feedback
Performance-related feedback	Intermittent feedback	Positive feedback after 3rd, 5th, 10th, 13th, 18th, 21st correct answer
	Constant feedback	Positive feedback after every correct answer
Performance-unrelated feedback	Constant feedback	Entertaining sound after every answer
Control condition	No-feedback	No feedback

The flanker task shows high re-test reliability (Intraclass correlations of.92; Bauer and Zelazo, 2014). Incongruent trials, in comparison to congruent trials, differentiate more precisely between subjects who are applying the task rule (i.e., respond to the orientation of the central fish) and those who are not applying the task rule. Thus, incongruent trials were used to calculate the dependent variable (Lee et al., 2013; Zelazo et al., 2013). There were two types of dependent variables: the percentage of correct answers and mean of the RT (for correct answers only).

### Control Variables

#### Passive Vocabulary

To rule out any systematic differences between the groups in terms SES, language skills were administered. The passive vocabulary test, a subtest from the Wechsler intelligence scale for preschool age (HAWIVA-III; Ricken et al., 2007), assessed participants’ language abilities. Out of four pictures, children had to choose the picture that matched the sentence read out by the experimenter. The dependent variable was the raw sum score (maximum total score = 31).

#### Visual Search Task

Attention is a prerequisite for goal maintenance as it enables the subject to focus on the goal (Marcovitch et al., 2007). Therefore, a visual search task served as a control measure to rule out systematic differences in selective attention (Breckenridge et al., 2013). On a white sheet of paper (A3 size, 297 mm × 420 mm) including 18 targets (18 red apples, each 1.5 × 1.5 cm) and 162 distractors (81 white apples and 81 red strawberries, each 1.5 × 1.5 cm), participants were given one minute to point out the targets. The experimenter marked all indicated items (targets and errors). The dependent variable was the number of correctly indicated targets.

### Design and Procedure

All tests were administered individually by trained experimenters. Within each age group, subjects were randomly assigned to one of the four feedback conditions. The order of the tasks was counterbalanced. Table 2 provides descriptive data for all variables.

**Table 2:** Descriptive statistics Standard deviations (SD) in parenthesis.

	3-year-olds				4-year-olds						
	**Experimental conditions**						
	**No FB^1^**	**Interm. FB^2^**	**Constant FB^3^**	**Perf. unrel. FB^4^**	**No FB^1^**	**Interm. FB^2^**	**Constant FB^3^**	**Perf. unrel. FB^4^**

*N*	21	19	21	23	16	19	18	22
Age, months	42 (3.0)	43 (2.8)	43 (2.8)	43 (3.1)	52 (3.8)	53 (3.9)	54 (3.0)	52 (3.1)
Female (%)	52	47	43	48	75	47	44	59
Passive Vocabulary	17.9 (3.2)	19.1 (3.3)	18.1 (4.4)	17.8 (3.7)	20.8 (2.8)	19.4 (4.0)	18.8 (3.5)	18.7 (5.1)
Visual search	6.4 (2.8)	6.6 (3.1)	6.0 (3.2)	5.8 (2.2)	9.5 (3.0)	9.6 (3.3)	8.7 (2.7)	7.6 (3.5)

^1^*no-feedback condition; ^*2*^intermittent feedback condition; ^*3*^constant feedback condition, ^*4*^performance-unrelated feedback condition*.

### Statistical Analysis

For the flanker task, RTs below 150 ms as well as RT exceeding the inter- and intra-individual mean by more than three standard deviations (SD) were considered as outliers and therefore excluded. In total, 2.3% of all data points were excluded. The mean accuracy score (ACC) was computed from the percentage of correct answers. All statistical analyses were run with SPSS (IBM, 2017, SPSS Statistics Version 25.0). As an estimator of effect sizes, partial eta^2^ values ($\eta_{p}^{2}$) are reported. *Post hoc* comparisons were obtained either by means of the Bonferroni test if homogeneity of variance was given, or by Games-Howell test if homogeneity of variance was violated.

The congruency effect is core feature of the flanker task. At the same time, the congruency effect provides evidence of increased inhibitory demands on incongruent trials compared to congruent trials (Ridderinkhof et al., 1997; Rueda et al., 2004). Analyses of variances (ANOVAs) for repeated measures confirmed the congruency effect: There was a significant main effect for congruency in terms of ACC [*F*(1,155) = 38.5, *p* < 0.000, $\eta_{p}^{2}$ = 0.20] and in terms of RT [*F*(1,155) = 33.2, *p* < 0.000, $\eta_{p}^{2}$ = 0.18]. The same pattern was also confirmed for each age group separately.

To exclude the possibility of systematic differences between the four conditions one-way ANOVAs were run for age, verbal ability and selective attention. Within each age group, no age differences were found across the four conditions [*F*_3-yr-olds_(3,83) < 1, *p* = 0.56, $\eta_{p}^{2}$ = 0.03; *F*_4-yr-olds_(3,74) < 1, *p* = 0.44 $\eta_{p}^{2}$ = 0.04]. Across the four experimental conditions, no differences in terms of verbal ability [*F*_3-yr-olds_(3,83) < 1, *p* = 0.67, $\eta_{p}^{2}$ = 0.01; *F*_4-yr-olds_(3,74) < 1, *p* = 0.40 $\eta_{p}^{2}$ = 0.04] nor selective attention [*F*_3-yr-olds_: (3,79) < 1, *p* = 0.79, $\eta_{p}^{2}$ = 0.01; *F*_4-yr-olds_(3,74) = 1.8, *p* = 0.16 $\eta_{p}^{2}$ = 0.07] were found.

## Results

### Feedback Effects

**Figure 1:**
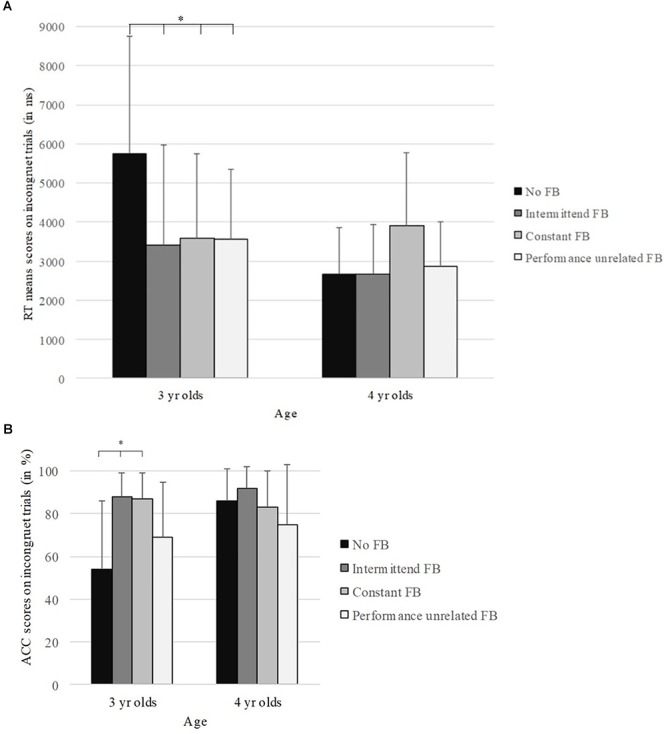
Flanker task means as a function of age group and condition, in terms of accuracy **(A)** and reaction time (RT) **(B)**. Significant differences between the no-feedback condition and the other feedback conditions are indicated with a ^∗^.

Figure 1 illustrates participants mean performance in the flanker task across different feedback conditions. Firstly, we tested if the level of success differed significantly from chance across the four conditions (see Table 3). Secondly, age-related performance differences across conditions were examined. An ANOVA was run with the between-subjects factors age (3-year olds, 4-year olds) and condition (no-feedback, intermittent feedback, constant feedback, performance-unrelated feedback). For both dependent variables (i.e., ACC and RT) the analysis revealed significant main effects: A main effect of age, ACC [*F*(1,158) = 8.2, *p* = 0.005, $\eta_{p}^{2}$ = 0.12] and RT [*F*(1,158) = 11.0, *p* = 0.001, $\eta_{p}^{2}$ = 0.07], and a main effect of condition, ACC [*F*(3,158) = 8.1, *p* < 0.000, $\eta_{p}^{2}$ = 0.14] and RT [*F*(3,158) = 2.7, *p* = 0.04, $\eta_{p}^{2}$ = 0.05]. The interaction between age and condition was significant too; ACC [*F*(3,158) = 5.2, *p* = 0.002, $\eta_{p}^{2}$ = 0.09] RT [*F*(3,158) = 4.9, *p* = 0.003, $\eta_{p}^{2}$ = 0.09]. Thirdly, to address feedback effects in more detail, ANOVAs were run separately for each age group. For the 3-year-olds, the analysis revealed a significant main effect of condition for both dependent variables, that is ACC, [*F*(1,83) = 10.9, *p* < 0.000, $\eta_{p}^{2}$ = 0.29], and RT, [*F*(1,83) = 4.5, *p* = 0.006, $\eta_{p}^{2}$ = 0.15]. Subsequent *post hoc* comparisons for ACC revealed significant differences between the no-feedback condition and the two performance-related conditions; intermittent feedback (*p* = 0.001), and constant feedback (*p* = 0.001). The difference between the no-feedback condition and the performance-unrelated feedback condition was not significant (*p* = 0.323). However, performance in the performance-unrelated feedback condition significantly differed from that in the performance-related feedback conditions, intermitted feedback (*p* = 0.020), and constant feedback (*p* = 0.024). Thus, subjects assigned to the performance-related feedback conditions responded more accurately compared to the subjects assigned to the no-feedback condition or the performance-unrelated feedback condition. For RT, *post hoc* comparisons revealed significant differences between the no-feedback condition and the three feedback conditions: intermittent feedback (*p* = 0.018), constant feedback (*p* = 0.029), and performance-unrelated feedback (*p* = 0.020). Thus, subjects responded faster in all three conditions with feedback compared to subjects assigned to the no-feedback condition (i.e., control condition). No differences were found between any of the feedback conditions.

**Table 3:** Accuracy performance tested against chance.

	*t*	df	*p*	CI
**3-year-olds**				
No FB	0.59	20	0.56	-0.10 –0.18
Intermitted FB	14.74	18	0.00	0.32 –0.43
Constant FB	13.98	20	0.00	0.32 –0.44
Perform. unrelated FB	3.55	22	0.00	0.08 –0.31
**4-year-olds**				
No FB	9.81	15	0.00	0.29 –0.45
Intermitted FB	17.98	18	0.00	0.37 –0.47
Constant FB	8.20	17	0.00	0.25 –0.42
Perform. unrelated FB	4.23	21	0.00	0.13 –0.38

*one-sample test, t-test value = 0.5*.

The identical analyses for the 4-year-olds revealed a significant main effect of condition for RT, [*F*(1,74) = 3.4, *p* = 0.02, $\eta_{p}^{2}$ = 0.13]. However, *post hoc* comparisons did not yield any significant differences between any of the feedback conditions: intermittent feedback (*p* = 1.00), constant feedback (*p* = 0.06), and performance-unrelated feedback (*p* = 1.00). For ACC, the main effect of condition was not significant [*F*(1,74) = 2.4, *p* = 0.07, $\eta_{p}^{2}$ = 0.09]. Thus, among the 4-year-olds, analyses did not reveal any reliable differences between conditions.

### Age Differences Across Feedback Conditions

The previous results revealed specific age effects: Performance of the 3-year-olds’ was superior in the conditions with feedback, whereas the 4-year-olds’ performance was largely unaffected by feedback. Therefore, age differences across the conditions were addressed in the final analysis. For every feedback condition, separate ANOVAs with age as between-subjects factor were calculated. For the no-feedback condition, there was a significant main effect of age in terms of ACC [*F*(1,36) = 14.3, *p* = 0.001, $\eta_{p}^{2}$ = 0.29], and in terms of RT [*F*(1,36) = 15.0, *p* < 0.001, $\eta_{p}^{2}$ = 0.30]. There were no main effects of age for any of the other feedback conditions: Intermittent feedback, ACC [*F*(1,37) = 1.3, *p* < 0.26, $\eta_{p}^{2}$ = 0.03], RT [*F*(1,37) = 1.4, *p* = 0.25, $\eta_{p}^{2}$ = 0.04]; constant feedback, ACC [*F*(1,38) < 1, *p* = 0.39, $\eta_{p}^{2}$2 = 0.02], RT [*F*(1,38) < 1, *p* = 0.62 $\eta_{p}^{2}$ = 0.01], or performance-unrelated feedback, ACC [*F*(1,44) < 1, *p* = 0.44, $\eta_{p}^{2}$ = 0.01], RT [*F*(1,44) = 2.3, *p* = 0.14, $\eta_{p}^{2}$ = 0.05].

## Discussion

The present study investigated if feedback can reduce goal-maintenance demands. There are two findings indicating that feedback affected goal maintenance in the flanker task: Firstly, 3-year-olds having received performance-related feedback outperformed those in the no-feedback condition in terms of accuracy by around 30%, and in terms of speed by around 2,000 ms. Secondly, accuracy performance of the 3-year-olds assigned to the no-feedback condition did not differ substantially from chance. This was not the case for any of the other conditions with feedback.

Therefore, we argue that goal-maintenance demands were reduced for the 3-year-olds, which in turn resulted in a more accurate and faster inhibitory performance. Applying the same line of argument for the 4-year olds, i.e., by comparing accuracy scores and means of RT across the four conditions, 4-year-olds’ goal maintenance did not seem dependent on feedback for that particular inhibition task. In fact, feedback did not affect performance, neither in terms of accuracy nor in terms of RT.

To examine if motivation would affect task performance, a performance-unrelated feedback condition was included. Performance for the performance-unrelated feedback condition was above chance, indicating that subjects were able to solve the task reliably with performance-unrelated feedback. Contrary to the no-feedback condition, for which performance was at chance level. The results suggest that performance-unrelated feedback affected task performance positively, most likely by tapping on motivational processes. Such a motivational based interpretation could also account for non-significant age differences between the 3-and 4-years-olds in the performance-unrelated feedback condition. However, the interpretation for motivation affecting task performance is not clear-cut due to two further findings: Firstly, *post hoc* comparisons between performance-related and performance-unrelated conditions showed reliable differences in favor of the performance-related feedback. Secondly, while *post hoc* comparisons between performance-related feedback and no-feedback showed substantial difference in terms of accuracy and speed, *post hoc* comparisons between performance-unrelated feedback and no-feedback showed only an effect for speed but not for accuracy. Thus, 3-year-olds assigned to the performance-unrelated feedback condition performed only faster but not more accurately compared to the no-feedback condition. Based on previous findings, we assume that both performance-related and performance-unrelated feedback increased inhibitory performance due to an increase in motivation (Dowsett and Livesey, 2000). However, only performance-related feedback further increased inhibitory performance due to decreased goal-maintenance demands. This might be due to the causal relation between positive feedback and *correct* performance affecting goal-maintenance demands of the task and would explain the pronounced inhibitory performance for the performance-related feedback conditions.

Another possible explanatory variable could be attention. It might be that regardless whether feedback was performance related or not, feedback affected the attentional system, supporting subjects in being more focused and alert on the task itself (Marcovitch et al., 2007), which in turn could explain the response speed findings (i.e., increased speed performance for the performance-unrelated feedback compared to the no-feedback condition). However, this is speculative. Further research is needed, manipulating not only goal-maintenance, but also attentional demands to examine the intertwined relation between motivation, attention and inhibition.

Frequency differences between the two performance-related feedback conditions (i.e., constant feedback condition, intermittent feedback condition) did not seem to impact performance. To examine performance progression across the trials, additional spilt half analyses for the two performance-related feedback conditions were run. There were no differences between the first and second half of the trials. Thus, although previous research suggests that constant feedback leads to a performance decline over time (Hattie and Timperley, 2007), no such effects over the course of 24 trials were found in the current study. Nevertheless, we would not exclude the possibility of constant feedback having a negative effect on performance. Instead, further research with larger numbers of trials and corresponding feedback would be necessary to examine possible negative effects of feedback on performance in the flanker task.

Performance between the two age groups differed only for the no-feedback condition. For the conditions with feedback, age differences were not significant. This means that with feedback, 3-year-olds were performing approximately on the same level as the 4-year-olds. A possible interpretation of this finding would be that 3- and 4-year-olds do not differ in terms of their inhibitory skills but instead differ greatly in terms of goal maintenance and to some extent motivation affecting task performance.

Despite the lack of feedback effects in the performance of the 4-year-olds, we would not conclude that 4-year-olds’ goal-maintenance skills cannot be affected by means of feedback. On a descriptive level, results were similar to the ones of the 3-year-olds. However, for the 4-year-olds, differences between the experimental conditions did not reach statistical significance. Nevertheless, it is possible that children at this age would benefit from feedback in a more challenging task. In addition, the rather small sample sizes might have made it more difficult to detect substantial differences between the conditions. In fact, the sample size might be the reason why no beneficial effects from feedback were found for the RT variable either. Nevertheless, we did find effects for the 3-year-olds, despite the small sample size. Still, future research should not only include a larger sample but also more difficult versions of the flanker task or more difficult inhibitory tasks to examine if feedback is an appropriate way to reduce goal-maintenance demands for older preschoolers.

Depending on the research question, there are different options to analyze flanker task data (e.g., sum scores, congruent-incongruent interference scores, or incongruent mean scores). Following previous research using the flanker task as a measure of inhibition (e.g., Lee et al., 2013; Zelazo et al., 2013; von Suchodoletz et al., 2017), we used incongruent mean scores. For the present study, this was especially expedient since feedback affected performance on congruent and incongruent trials differently. More precisely, feedback effects were more pronounced for incongruent trials than for congruent trials, likely because children were already performing quite well on congruent trials, leaving less possibility for improvement via feedback. Under the premise that feedback affects congruent trials and incongruent trials similarly, future studies using a more difficult version of the flanker task could apply and compare different analyses options.

To wrap up, previous research has shown that goal-maintenance demands can be reduced directly by means of transparent cues associated with task goal (Rueda et al., 2004; Chevalier and Blaye, 2008; Blaye and Chevalier, 2011). With the present study, we aimed to extend this approach by reducing goal maintenance demands indirectly by means of feedback. For the 3-year olds, the present results revealed similar findings to previous research: Through indirect manipulation of goal maintenance - by means of feedback - inhibitory performance increased. Although the precise effect of feedback on motivation has yet to be established, the results show a specific benefit for inhibitory performance if feedback is performance-related. As previous work has already pointed out (Ridderinkhof et al., 1997; Bub et al., 2006), the present findings underline the importance to consider and control for goal maintenance before drawing conclusion on participants’ inhibition skills.

In everyday life, demands on inhibition and goal maintenance rarely occur isolated. However, distinguishing between the two seems relevant not only for theoretical purposes, but also for practical ones. From a theoretical perspective, unraveling inhibition and goal maintenance contributes to a better understanding of the inhibition construct. From a practical perspective, distinguishing precisely between inhibition and goal maintenance might be important for conducting appropriate interventions. Whenever a child shows inhibitory deficits, it may be worth considering his or her goal-maintenance skills too, to prevent the effects of goal-maintenance deficits cascading across different cognitive domains.

## Ethics Statement

This study was carried out in accordance with the recommendations of “Ethikkommission der Philosophisch-humanwissenschaftliche Fakultät der Universität Bern” with written informed consent from all partents and verbal consent from all children. All subjects gave written informed consent in accordance with the Declaration of Helsinki. The protocol was approved by the “Ethikkommission der Philosophisch-humanwissenschaftliche Fakultät der Universität.”

## Author Contributions

All authors contributed to the conception and design of the study. NO organized the database. DB and NO performed the statistical analysis. NO wrote the first draft of the manuscript. CR and DB critically revised the draft. CR, DB, and NO contributed to manuscript revision, read and approved the submitted version.

## Conflict of Interest Statement

The authors declare that the research was conducted in the absence of any commercial or financial relationships that could be construed as a potential conflict of interest.
